# Results of a pilot study using self-collected mid-turbinate nasal swabs for detection of influenza virus infection among pregnant women

**DOI:** 10.1111/irv.12309

**Published:** 2015-04-23

**Authors:** Mark G Thompson, Jeannette R Ferber, Roxana Odouli, Donna David, Pat Shifflett, Jennifer K Meece, Allison L Naleway, Sam Bozeman, Sarah M Spencer, Alicia M Fry, De-Kun Li

**Affiliations:** a Influenza Division, Centers for Disease Control and Prevention (CDC)Atlanta, GA, USA; b Division of Research, Kaiser Foundation Research InstituteOakland, CA, USA; c Marshfield Clinic Research FoundationMarshfield, WI, USA; d Abt Associates, Inc.Cambridge, MA, USA; e Center for Health Research, Kaiser Permanente NorthwestPortland, OR, USA

**Keywords:** Acute respiratory illness, influenza, nasal swab, pregnancy, self-collection

## Abstract

**Background:**

We evaluated the feasibility of asking pregnant women to self-collect and ship respiratory specimens.

**Methods:**

In a preliminary laboratory study, we compared the RT-PCR cycle threshold (CT) values of influenza A and B viruses incubated at 4 storage temperatures (from 4 to 35°C) for 6 time periods (8, 24, 48, 72, and 168 hours and 30 days), resulting in 24 conditions that were compared to an aliquot tested after standard freezing (−20°C) (baseline condition). In a subsequent pilot study, during January–February, 2014, we delivered respiratory specimen collection kits to 53 pregnant women with a medically attended acute respiratory illness using three delivery methods.

**Results:**

CT values were stable after storage at temperatures <27°C for up to 72 hours for influenza A viruses and 48 hours for influenza B viruses. Of 53 women who received kits during the pilot, 89% collected and shipped nasal swabs as requested. However, 30% (14/47) of the women took over 2 days to collect and ship their specimen. The human control gene, ribonuclease P (RNase P), was detected in 100% of nasal swab specimens. However, the mean CT values for RNase P (26·5, 95% confidence interval [CI] = 26·0–27·1) and for the 8 influenza A virus positives in our pilot (32·2, 95% CI = 28·9–35·5) were significantly higher than the CTs observed in our 2010–2012 study using staff-collected nasal pharyngeal swabs (*P*-values < 0·01).

**Discussion:**

Self-collection of respiratory specimens is a promising research method, but further research is needed to quantify the sensitivity and specificity of the approach.

Household- and community-based studies of the epidemiology and prevention of influenza virus infection are rare, in large part because of their high costs and many logistical challenges.^1^,^2^ In our 2010–2012 study of influenza vaccine effectiveness among pregnant women,^3^ trained staff collected respiratory specimens from over 300 women by visiting their homes, which were spread out over 500 square miles in two metropolitan areas on the USA West Coast. Encouraged by the results of several studies that examined self-collected respiratory specimens,^4^–^9^ we conducted a follow-up study in 2013–2014 to explore the feasibility of forgoing household visits by asking pregnant women with acute respiratory illness (ARI) to self-collect and ship nasal swabs.

We began by conducting a storage time and temperature laboratory experiment to determine the stability of influenza virus exposed to a range of temperatures over several time windows. Next, we delivered respiratory specimen collection kits to pregnant women with ARI using three delivery methods and recorded if and when they completed the task. Finally, we examined the overall quality of specimens collected by comparing the detection of human nucleic acid and influenza A virus from specimens self-collected with nasal swabs versus specimens staff-collected using nasal pharyngeal (NP) swabs in our previous 2010–2012 study.^3^,^10^

## Methods

### Laboratory evaluation of the effects of storage time and temperature on influenza virus detection

At the study reference laboratory (Marshfield Clinic Research Foundation laboratory in Marshfield, Wisconsin), four influenza isolates from specimens originally collected in 2012–2013 (A/CALIFORNIA/07/2009-like[H1N1]pdm09, A/VICTORIA/361/2011-like A[H3N2], B/WISCONSIN/01/2010-like B/Yamagata, and B/BRISBANE/60/2008-like B/Victoria) with a hemagglutination inhibition (HI) of 1:64 were diluted 1:1000 with M4-RT® transport medium (Remel, Lenexa, KS, USA). Each of the diluted viruses was divided into 25 aliquots of 500 μl each. Human buccal cells were added to each of the influenza isolates for the endogenous control gene ribonuclease P (RNase P). One aliquot per virus was incubated at 4 storage temperatures (4°C, room temperature of ∼20, 27, and 35°C) for 6 time periods (8, 24, 48, 72, and 168 hours and 30 days), resulting in 24 conditions that were compared to an aliquot tested after standard freezing (−20°C) (baseline condition) (Table S1).

Total nucleic acid isolation and purification was performed using automated magnetic bead technology (MagNA Pure LC.2.0 system, Roche Diagnostics, Indianapolis, Indiana). Real-time reverse transcription PCR (rRT-PCR) was performed on nucleic acid extracts using a LightCycler Real-Time PCR System (Roche Diagnostics, Basel, Switzerland). The rRT-PCR assay is a TaqMan®-based real-time detection of the matrix protein (M1) of influenza A and the non-structural protein 1 (NS1) of influenza B. Centers for Disease Control and Prevention (CDC) primers, probes, and procedures were used.

The rRT-PCR cycle threshold (CT) values indicate when fluorescence generated in the reaction significantly exceeds background and are inversely proportional to the amount of nucleic acid target that is present in the specimen.^11^,^12^ CT values for detection of each virus and RNase P were compared with the baseline CT. We considered as noteworthy an absolute difference of >1 CT value or a >5% change.

### Pilot test of self-collection and shipping of respiratory specimens

Participants were pregnant members of the Kaiser Permanente Northern California (KPNC) health plan (San Francisco Bay Area) who had attended at least one prenatal visit. As described previously,^3^,^10^ we identified potential ARIs using daily surveillance of electronic medical records for medically attended acute respiratory illness (MAARI) (using ICD-9-CM codes 460–466, 480–488, and 490–91). Screening for an illness with fever and cough (with onset ≤8 days) was completed by telephone.

Participants with MAARI who provided verbal consent were randomly assigned to one of three approaches to delivering specimen collection kits to their home: (i) Kits were delivered by study staff who were available to answer questions, (ii) kits were delivered by a local courier, and (iii) kits were delivered by next-day mail (limited to participants enrolled prior to 3:00 p.m. Monday–Thursday).

Participants were given written instructions and directed to view an online video (Appendix S1), which demonstrated how to collect a respiratory specimen using a mid-turbinate nasal swab, place the swab in a tube containing M4-RT transport medium, and ship the specimen along with a signed consent form using the packaging provided (without freezing or cold packs). Participants were asked to collect specimens “as soon as possible” and then contact a commercial shipping service [United Postal Service (UPS)] directly for package pickup at their home or drop off the package at a UPS drop-box for next-day delivery to the laboratory.

Each participant was offered a small incentive in the form of a gift card. Study procedures were reviewed and approved by the KPNC and Abt Associates institutional review boards.

### Evaluation of specimen quality

Specimens were shipped to the reference laboratory via UPS. Using rRT-PCR as described above, a CT <40·0 for the endogenous control gene, RNase P, indicted detection of human nucleic acid, while CT ≥40 indicated that the specimen was of poor quality and/or quantity. Although CDC's influenza rRT-PCR protocol does not quantify viral load in respiratory specimens, CT values have been used to estimate the relative amounts of influenza virus present in studies of influenza severity and evaluations of influenza treatments.^10^,^13^–^15^ We compared the mean and 95% confidence intervals (CI) of CT RNase P for specimens self-collected in this study and for 292 specimens from our 2010 to 2012 study,^3^,^10^ which involved staff-collected NP specimens tested in the same laboratory with the same equipment and procedures. We also compare the CT values of influenza A positives identified in the pilot with CTs observed for 79 influenza A positives from the 2010 to 2012 study. Mean CT values were estimated using a generalized linear model; influenza A CT values were estimated adjusting for days from illness onset since swabbed, as influenza viral shedding declines with time^16^–^18^; a difference between estimates with *P* < 0·01 was considered statistically significant.

## Results

### Influenza detection after different storage times and temperatures

**Figure 1:**
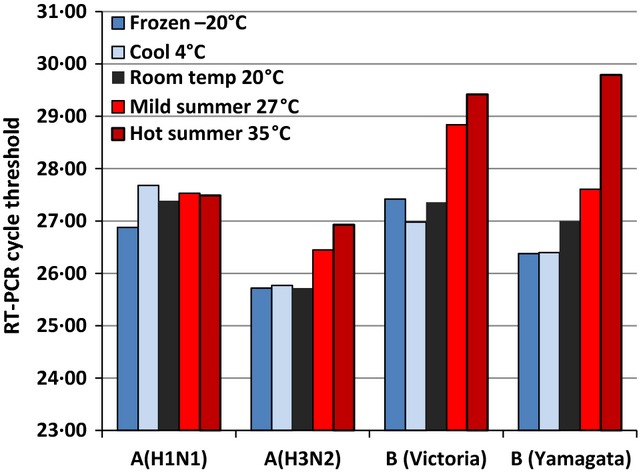
Cycle threshold from rRT-PCR of four influenza viruses stored for 72 hours at five temperatures.

We observed little change in rRT-PCR CT values for the influenza A viruses tested following up to 72 hours in 4 storage temperatures (Table S1A). At room temperature (20°C), CT values for the influenza A viruses increased by less than 5% following exposure for up to 168 hours and by ∼9% after 30 days. A consistent trend we noted across influenza A and B viruses was an elevation in CT values (lower target load) by 5% or by 1 CT at the higher temperatures (27 and 35°C) after a 168-hour delay or longer. For the two influenza B viruses, we noted elevation in CT values at higher temperatures after only a 48-hour delay. Figure1 illustrates the CT values observed for each of the influenza viruses at the different storage temperatures after 72 hours. For the endogenous control gene RNase P, CT values remained largely stable across temperature conditions for up to 168 hours, but were elevated for aliquots stored for 30 days (Table S1B).

### Completion of self-collection and shipping of respiratory specimens

We identified 378 pregnant women through MAARI surveillance during January and February, 2014; 262 were reached by telephone (69%). Of those contacted, 89 (34%) reported an acute illness with cough and fever; 9 (10%) of these women refused to participate, and 27 (30%) were excluded because their illness onset was >8 days. Among the remaining 53 eligible participants, 36% were in their first trimester, 38% in their second, and 26% in their third trimester. The mean age was 31 years old (range = 18–43 years); the race/ethnicity of participants was Caucasian (43%), Asian (18%), or Black (14%); 30% were Hispanic. Most (64%) had attended one or more years of college, and most women described their overall health as very good (39%) or excellent (34%).

The vast majority of women (89%) collected the respiratory specimen and shipped it to the laboratory; the response was similar across the three delivery methods we piloted (Table1).

**Table 1:** Self-collection of respiratory specimens, delays in shipping, and gap between illness onset and shipping among a pilot sample of 53 pregnant women

Collected and shipped respiratory specimen?	Not shipped	shipped
Specimen kit delivered by study staff (*n* = 20)		18 (90%)
Failed to ship within 7 days	1 (5%)	
Shipped vial but without swab	1 (5%)	
Specimen kit delivered by courier (*n* = 22)		20 (90%)
Failed to ship within 7 days	1 (5%)	
Never shipped specimen	1 (5%)	
Specimen kit mailed to participants (*n* = 11)*		9 (82%)
Never shipped specimen	2 (18%)	

Delay (in days) from receipt of specimen kit to shipping?†	(Min, Max)	Median	≤2 days
Specimen kit delivered by study staff (*n* = 18)	(0, 4)	2	12 (67%)
Specimen kit delivered by courier (*n* = 20)	(0, 7)	1	15 (71%)
Specimen kit mailed to participants (*n* = 9)	(0, 4)	1	6 (67%)

Among women screened ≤6 days from illness onset, gap (in days) from illness onset to specimen shipping?	(Min, Max)	Median	≤8 days
Specimen kit delivered by study staff (*n* = 9)	(4, 8)	5	9 (100%)
Specimen kit delivered by courier (*n* = 11)	(4, 8)	6	11 (100%)
Specimen kit mailed to participants (*n* = 8)	(3, 11)	5·5	6 (75%)

* Only participants enrolled before 3 p.m. Monday through Thursday could be randomized to the mailed kit method, in order to meet next-day delivery deadlines. Thus, the number assigned to this group is approximately half that of the other two methods.

† Delivery date is considered day zero; the next day is day 1.

Only 17% of the women (8/47) collected and shipped the specimen on the same day when it was delivered. As part of the first delivery method, study staff offered to wait ∼20 minutes and pickup the specimen kit directly from participants; however, only 2 of 18 women accepted this offer. Across the delivery methods we piloted, most women waited at least until the following day, and 70% (33/47) shipped the specimen within 2 days.

Most women had been ill for several days when screened (median = 6 days); therefore, a delay of an additional 1 or 2 days meant that 32% (15/47) had been ill for >8 days when their specimens were shipped to the laboratory. If we had narrowed eligibility to women who had been ill for ≤6 days at time of screening, it would have excluded over one-third of the participants (19/47, 40%). However, narrowing participants to those ill ≤6 days would have allowed an additional 1 or 2 days to collect and ship specimens. Indeed, the vast majority of women in our pilot who had been sick ≤6 days (26/28, 93%) were able to collect and ship their specimen within 8 days of illness onset.

### Quality of self-collected respiratory specimens compared with staff-collected specimens

**Figure 2:**
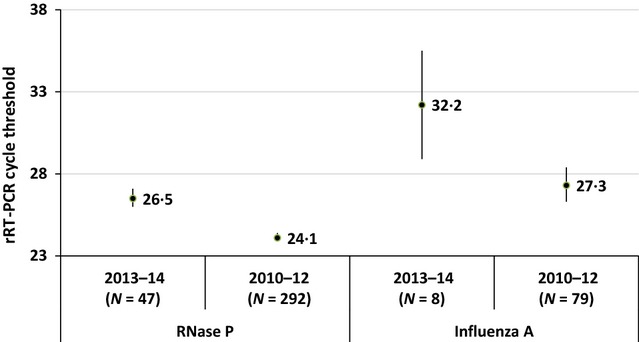
Cycle thresholds (and 95% confidence intervals) from rRT-PCR of ribonuclease P (RNase P) and influenza A viruses collected from pregnant women with acute respiratory illness during a pilot study of nasal swab self-collection (2013–2014) and a prior study with staff-collected nasal pharyngeal swabs (2010–2012).

Human nucleic acid was detected in 100% of the 47 self-collected nasal swab specimens tested; the CT for RNase P ranged from 22·7 to 31·3 with a mean of 26·5 (95% CI = 26·0–27·1). These CT values are significantly higher (indicating lower target load) than the CT values for RNase P observed for 292 specimens from staff-collected NP swabs which ranged from 19·8 to 31·5 with a mean of 24·1 (95% CI = 23·9–24·4) (Wald χ^2^ [1] = 61·0, *P* < 0·0005) (Figure2).

In our current pilot test during the 2013–2014 influenza season, 9 of 47 (19%) women with MAARI were rRT-PCR positive for influenza A or B virus infections; in contrast, 100 of 292 (34%) women during our 2010–2012 study were influenza positive. Among the 8 influenza A positives in the pilot (6 A[H1N1]pdm09 and 2 unsubtyped A viruses), the CT for the A virus assay ranged from 23·3 to 37·5 with a mean CT (adjusted for days since illness onset) of 32·2 (95% CI = 28·9–35·5). This was significantly higher than the CTs observed for 79 influenza A positives from the 2010 to 2012 study (47 A[H1N1]pndm09, 34 A[H3N2]), which ranged from 14 to 38 with an adjusted mean CT of 27·3 (95% CI = 26·3–28·4) (Wald χ^2^ [1] = 7·6, *P* = 0·006) (Figure2). Indeed, 28% of the influenza A CT values from our original study were below 23 (the lowest CT value from the current pilot study). In a model that adjusted for the CT of RNase P in each specimen, the difference in influenza A CT values between the pilot study and our earlier study declined (mean CT of 30·0 and 27·5, respectively) and was no longer statistically significant (Wald χ^2^ [1] = 1·7, *P* = 0·20). Very similar results were observed when we limited viruses to A(H1N1)pdm09, which was a common virus across seasons (data not shown).

## Discussion

Encouraged by findings from our laboratory experiment which suggested influenza viruses would remain stable during a shipping period of up to 3 days for influenza A viruses and 2 days for influenza B viruses at room temperature or cooler (and thus not require cold chain management), we piloted sending respiratory specimen collection kits to the homes of 53 pregnant women with medically attended ARI. Although 9 out of 10 participants collected and shipped nasal swabs as requested and 100% of specimens had detectable RNase P, rRT-PCR CT values for RNase P and CT values for influenza A virus detection were significantly higher in our pilot compared with CTs observed in our 2010–2012 study using staff-collected NP swabs. Although the magnitude of the CT differences (27 versus 24 for RNase P and 32 versus 27 for influenza A, respectively) were modest, further research is needed to determine the implications for applying self-collection methods and interpreting rRT-PCR results.

Nonetheless, we drew four conclusions from our laboratory and pilot studies. First, self-collection and shipping of respiratory specimens is feasible. Only 11% of consented participants failed to ship specimens as instructed, and we had similar success regardless of how the specimen kits were delivered, which indicates the flexibility of the method. Our experience adds to similar encouraging findings regarding the feasibility of nasal swab self-collection among adults with respiratory illness.^5^,^7^,^9^ In fact, the level of participation in our study was much higher than these previous studies, which is especially encouraging given that pregnancy is often a very busy and stressful time in women's lives.^19^

Second, the method appears best suited for studying ARIs shortly after illness onset, as most nasal swabs were collected and shipped 1–2 days after kit delivery. This is similar to delays noted in a surveillance study in the United Kingdom.^5^ We expect the turnaround time for swab self-collection and shipping could have been improved through better communication at enrollment and reminders after the kit was delivered. Nonetheless, the timely collection of respiratory specimens is already a significant challenge for influenza researchers, as viral shedding declines with longer illness duration.^16^–^18^ In our pilot study which contacted women after a medical encounter, one-third of the consented women were excluded because their illnesses began >8 days earlier. Further restricting ARIs to ≤6 days since illness onset would exclude an additional one-third. Therefore, if this method further restricts eligibility, more effort will have to go into recruitment to offset these exclusions. Bias may also be introduced if individuals who seek medical care later in their illness differ from early care seekers in their underlying health or propensity to be vaccinated.^20^

Third, assuming that the findings from our laboratory experience would apply to viruses collected outside of a laboratory, it appears that our pilot methodology, which shipped specimens without refrigeration or cold chain management during wintertime in a temperate climate, was adequate. In fact, we continued to detect both A and B influenza viruses in our laboratory study after storage at very high temperatures and even after 30 days. Nonetheless, in our experiment, the ease of detecting influenza viruses (especially B viruses) declined slightly with longer storage times at warmer temperatures; thus, further research is needed to explore whether self-collection and shipping is sufficient during other seasons in temperate climates or in tropical climates.

Fourth, similar to other recent studies,^6^ we found the quality of respiratory specimens from self-collected nasal swabs appears to be adequate. None of the self-collected specimens had to be rejected due to poor quality and/or quantity. Our ability to detect human nucleic acid was modestly lower using specimens self-collected with nasal swabs compared to specimens collected by trained staff using a more invasive NP swab. Both methods also identified influenza virus infections using rRT-PCR, which is a sensitive assay. However, the CTs of the rRT-PCR detection of influenza A virus infections using specimens from staff-collected NP swabs in our 2010–2012 study were lower than the CTs observed in our pilot study, suggesting it was somewhat more difficult (or took longer) to detect influenza viruses using self-collected specimens.

The reason for this is unclear. Differences in swab type may play a role.^7^,^21^ As the difference we observed declined when we adjusted for RNase P, it is possible that influenza detection was impaired by somewhat lower specimen quality.^22^ Our pilot participants and those in our 2010–2012 studies also differed in the days since illness onset when swabbed. Although we adjusted for this statistically, residual bias may remain. Other possible factors include differences in circulating strains, collection technique, specimen management and shipping, or a combination of factors. The magnitude and effect of any bias is also unclear, as we do not know how many true infections were missed using either method.

Strengths of our study include the use of rRT-PCR to examine the stability of influenza virus detection, to quantify specimen quality, and to compare self-collected versus staff-collected respiratory specimens. The generalizability of our findings is enhanced by our recruitment of participants using prospective surveillance in contrast to previous self-swabbing pilots that relied on convenience samples.^6^,^7^,^9^ By randomly assigning participants to three delivery methods, we increased our confidence that the acceptability of the method is not limited to a specific delivery approach. Our focus on pregnant women also adds to the value of our pilot, as innovative community-based methods are especially needed to address many unanswered questions about the epidemiology of influenza and vaccine effectiveness among pregnant women.^3^,^10^,^23^

In addition to the limitations already mentioned, our study has at least five other limitations. First, our laboratory experiment only examined the effects of consistent storage temperatures on aliquoted specimens; further research is needed to assess the effects of temperature fluctuations on participant-collected swabs stored prior to aliquoting and in more real-world scenarios, such as repeated freezing and thawing, which may occur during wintertime shipping. Second, we assessed ARI symptoms at enrollment but did not document symptoms when self-swabbing occurred, which was typically 2 days later. Although the average woman in our 2010–2012 study was sick for an additional 5 days following specimen collection,^10^ we do not know the extent to which illnesses had improved by the time of self-swabbing or whether this influenced our results. Third, we do not know the reasons why some women significantly delayed or failed to ship specimens. Further research is needed on the knowledge, attitudes, and beliefs of participants after receiving self-collection instructions and on ways to improve adherence and timely response. Fourth, we did not quantify the relative costs of the self-collection method compared to our earlier method that involved home visits by staff. However, we agree with other study designers 4,5,24 that the savings are likely to be substantial. Fifth, the extent to which our findings regarding the feasibility of self-swabbing would generalize beyond an insured and mostly college-educated population is unclear.

In summation, self-collection of respiratory specimens is a promising research method that may reduce research costs, minimize secondary exposure of viruses to research staff (especially if faced with a highly infectious novel or pandemic virus), and expand the settings, populations, and contexts in which influenza can be studied. Given the logistical and funding challenges to enrolling a sufficient number of influenza-positive cases for influenza vaccine effectiveness research,^2^,^25^ the primary value of self-swabbing studies may be in their capacity to identify more influenza positives. This will likely come at the cost of adding false influenza negatives to the controls. Any method of respiratory specimen collection, especially those using a single swab type, may miss some influenza infections that would have been identified using other swab types or combinations.^7^,^15^ Further research is needed to quantify the sensitivity and specificity of the self-collection approach and to inform the trade-offs researchers must make in designing community-based influenza studies.

## Conflicts of interest

None reported.

## Supporting Information

**Table S1A.** Cycle threshold (CT) from RT-PCR of four influenza viruses at six time periods of delay prior to testing and four storage temperatures with change in CT and percentage [%] change from baseline.

**Table S1B.** Cycle threshold (CT) from RT-PCR of Rnase P endogenous control gene, at six time periods of delay prior to testing and four storage temperatures with change in CT and percentage [%] change from baseline.

**Appendix S1.** Swab self-collection instructions.
