# Supplementary material for: Results of a pilot study using self-collected mid-turbinate nasal swabs for detection of influenza virus infection among pregnant women
Source: Influenza Other Respir Viruses. 2015 Apr 23;9(3):155–60. doi: 10.1111/irv.12309 (PMC4415700; doi:10.1111/irv.12309)
Supplement: Supplementary file 1 [file irv0009-0155-sd1.pdf]

Supplemental Table A. Cycle threshold (CT) from RT-PCR of four influenza viruses at six time periods of delay prior to testing and four storage temperatures with change in CT and percentage [%] change from baseline

Time Delay	Storage Temperature	A(H1N1)pdm09 Virus			A(H3N2) Virus			B/Victoria Virus			B/Yamagata		
		CT	CT Change	% Change	CT	CT Change	% Change	CT	CT Change	% Change	CT	CT Change	% Change
Baseline (none)	-20°C	26.88			25.72			27.42			26.38		
8 Hours	4°C	26.57	0.31	1.15	24.95	0.77	2.99	26.94	0.48	1.75	26.75	-0.37	-1.40
	20°C	26.74	0.14	0.52	25.73	-0.01	-0.04	27.74	-0.32	-1.17	26.49	-0.11	-0.42
	27°C	26.77	0.11	0.41	25.88	-0.16	-0.62	27.26	0.16	0.58	26.81	-0.43	-1.63
	35°C	26.90	-0.02	-0.07	26.40	-0.68	-2.64	27.67	-0.25	-0.91	26.86	-0.48	-1.82
24 Hour	4°C	26.70	0.18	0.67	25.93	-0.21	-0.82	26.60	0.82	2.99	26.38	0.00	0.00
	20°C	26.91	-0.03	-0.11	25.38	0.34	1.32	26.83	0.59	2.15	26.47	-0.09	-0.34
	27°C	26.73	0.15	0.56	25.62	0.10	0.39	27.57	-0.15	-0.55	26.85	-0.47	-1.78
	35°C	27.23	-0.35	-1.30	26.52	-0.80	-3.11	27.86	-0.44	-1.60	27.58	-1.20	-4.55
48 Hour	4°C	26.76	0.12	0.45	25.94	-0.22	-0.86	26.91	0.51	1.86	26.17	0.21	0.80
	20°C	27.28	-0.40	-1.49	25.65	0.07	0.27	26.80	0.62	2.26	26.69	-0.31	-1.18
	27°C	26.91	-0.03	-0.11	25.74	-0.02	-0.08	27.99	-0.57	-2.08	27.35	-0.97	-3.68
	35°C	27.28	-0.40	-1.49	26.54	-0.82	-3.19	28.57	-1.15	-4.19	28.51	-2.13	-8.07
72 Hour	4°C	27.68	-0.80	-2.98	25.77	-0.05	-0.19	26.98	0.44	1.60	26.40	-0.02	-0.08
	20°C	27.39	-0.51	-1.90	25.72	0.00	0.00	27.36	0.06	0.22	27.00	-0.62	-2.35
	27°C	27.53	-0.65	-2.42	26.45	-0.73	-2.84	28.84	-1.42	-5.18	27.61	-1.23	-4.66
	35°C	27.49	-0.61	-2.27	26.93	-1.21	-4.70	29.42	-2.00	-7.29	29.79	-3.41	-12.93
168 Hour	4°C	26.59	0.29	1.08	26.32	-0.60	-2.33	27.23	0.19	0.69	26.65	-0.27	-1.02
	20°C	27.36	-0.48	-1.79	26.67	-0.95	-3.69	27.94	-0.52	-1.90	27.70	-1.32	-5.00
	27°C	27.93	-1.05	-3.91	26.69	-0.97	-3.77	29.77	-2.35	-8.57	28.91	-2.53	-9.59
	35°C	28.67	-1.79	-6.66	27.44	-1.72	-6.69	30.84	-3.42	-12.47	30.90	-4.52	-17.13
30 Days	4°C	28.33	-1.45	-5.39	27.27	-1.55	-6.03	28.51	-1.09	-3.98	28.23	-1.85	-7.01
	20°C	29.40	-2.52	-9.38	27.95	-2.23	-8.67	31.91	-4.49	-16.37	30.82	-4.44	-16.83
	27°C	30.21	-3.33	-12.39	30.26	-4.54	-17.65	33.70	-6.28	-22.90	32.33	-5.95	-22.55
	35°C	33.34	-6.46	-24.03	33.42	-7.70	-29.94	36.89	-9.47	-34.54	34.41	-8.03	-30.44

CT Change: Baseline Temp CT - Experimental Temp CT

% Change: (Baseline Temp CT - Experimental Temp CT)/Baseline Temp CT*100

Note: If the CP Change and % CP are negative values, the CP value INCREASED from the Baseline

Highlighted cells represent >1 absolute value CT change or a change of >5%.
