# Supplementary material for: Results of a pilot study using self-collected mid-turbinate nasal swabs for detection of influenza virus infection among pregnant women
Source: Influenza Other Respir Viruses. 2015 Apr 23;9(3):155–60. doi: 10.1111/irv.12309 (PMC4415700; doi:10.1111/irv.12309)
Supplement: Supplementary file 2 [file irv0009-0155-sd2.pdf]

Supplemental Table B. Cycle threshold (CT) from RT-PCR of Rnase P endogenous control gene, at six time periods of delay prior to testing and four storage temperatures with change in CT and percentage [%] change from baseline

Time Delay	Storage Temperature	A(H1N1)pdm09 Virus			A(H3N2) Virus			B/Victoria Virus			B/Yamagata		
		CT	CT Change	% Change	CT	CT Change	% Change	CT	CT Change	% Change	CT	CT Change	% Change
**Baseline (none)**	**-20°C**	29.44			29.58			30.30			30.40		
**8 Hours**	**4°C**	28.40	1.04	3.53	26.81	2.77	9.36	30.28	0.02	0.07	30.33	0.07	0.23
	**20°C**	29.12	0.32	1.09	29.51	0.07	0.24	29.86	0.44	1.45	29.82	0.58	1.91
	**27°C**	29.56	-0.12	-0.41	29.91	-0.33	-1.12	29.76	0.54	1.78	30.25	0.15	0.49
	**35°C**	30.31	-0.87	-2.96	29.53	0.05	0.17	30.52	-0.22	-0.73	29.89	0.51	1.68
**24 Hour**	**4°C**	29.52	-0.08	-0.27	29.64	-0.06	-0.20	29.43	0.87	2.87	29.39	1.01	3.32
	**20°C**	29.54	-0.10	-0.34	29.41	0.17	0.57	29.64	0.66	2.18	29.86	0.54	1.78
	**27°C**	29.66	-0.22	-0.75	29.16	0.42	1.42	29.93	0.37	1.22	29.64	0.76	2.50
	**35°C**	28.89	0.55	1.87	29.74	-0.16	-0.54	28.43	1.87	6.17	30.23	0.17	0.56
**48 Hour**	**4°C**	28.64	0.80	2.72	27.94	1.64	5.54	29.79	0.51	1.68	29.36	1.04	3.42
	**20°C**	28.88	0.56	1.90	28.27	1.31	4.43	29.52	0.78	2.57	29.80	0.60	1.97
	**27°C**	29.62	-0.18	-0.61	29.33	0.25	0.85	29.95	0.35	1.16	29.11	1.29	4.24
	**35°C**	30.19	-0.75	-2.55	30.60	-1.02	-3.45	29.96	0.34	1.12	30.71	-0.31	-1.02
**72 Hour**	**4°C**	29.83	-0.39	-1.32	28.40	1.18	3.99	29.66	0.64	2.11	30.65	-0.25	-0.82
	**20°C**	29.80	-0.36	-1.22	28.78	0.80	2.70	29.60	0.70	2.31	29.72	0.68	2.24
	**27°C**	29.66	-0.22	-0.75	29.73	-0.15	-0.51	30.50	-0.20	-0.66	30.07	0.33	1.09
	**35°C**	29.30	0.14	0.48	29.72	-0.14	-0.47	29.86	0.44	1.45	30.42	-0.02	-0.07
**168 Hour**	**4°C**	29.42	0.02	0.07	29.60	-0.02	-0.07	30.58	-0.28	-0.92	31.21	-0.81	-2.66
	**20°C**	30.27	-0.83	-2.82	29.82	-0.24	-0.81	30.83	-0.53	-1.75	31.70	-1.30	-4.28
	**27°C**	29.96	-0.52	-1.77	30.55	-0.97	-3.28	30.95	-0.65	-2.15	29.95	0.45	1.48
	**35°C**	30.22	-0.78	-2.65	31.34	-1.76	-5.95	30.42	-0.12	-0.40	30.77	-0.37	-1.22
**30 Days**	**4°C**	31.72	-2.28	-7.74	32.70	-3.12	-10.55	31.59	-1.29	-4.26	31.49	-1.09	-3.59
	**20°C**	31.91	-2.47	-8.39	30.81	-1.23	-4.16	32.24	-1.94	-6.40	31.50	-1.10	-3.62
	**27°C**	31.91	-2.47	-8.39	32.84	-3.26	-11.02	32.59	-2.29	-7.56	31.73	-1.33	-4.38
	**35°C**	33.75	-4.31	-14.64	33.26	-3.68	-12.44	31.83	-1.53	-5.05	33.15	-2.75	-9.05

CT Change: Baseline Temp CT - Experimental Temp CT
% Change: (Baseline Temp CT - Experimental Temp CT)/Baseline Temp CT*100
Note: If the CP Change and % CP are negative values, the CP value INCREASED from the Baseline
Highlighted cells represent >1 absolute value CT change or a change of >5%.
