# Supplementary material for: Results of a pilot study using self-collected mid-turbinate nasal swabs for detection of influenza virus infection among pregnant women
Source: Influenza Other Respir Viruses. 2015 Apr 23;9(3):155–60. doi: 10.1111/irv.12309 (PMC4415700; doi:10.1111/irv.12309)
Supplement: Supplementary file 3 [file irv0009-0155-sd3.pdf]

Appendix

Written instructions for method involving delivery by study staff (attached)

Video instructions for study staff method

http://www.youtube.com/watch?v=BV2ril3a5DY

Written instructions for mailed kit method (attached)

Video instructions for mailed kit method

http://www.youtube.com/watch?v=RkSZaHjJ1nc

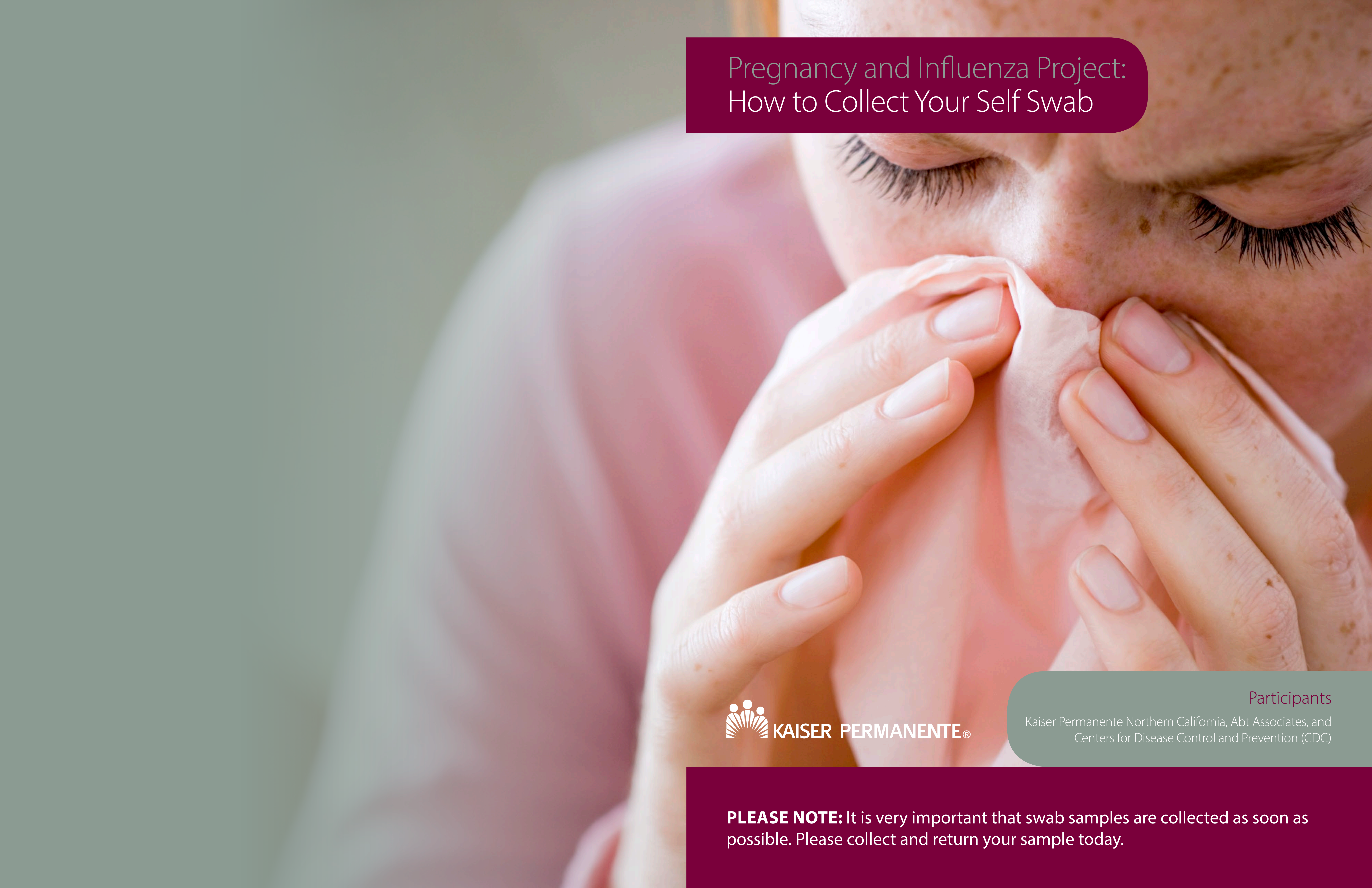

Pregnancy and Influenza Project:
How to Collect Your Self Swab

KAISER PERMANENTE®

Participants

Kaiser Permanente Northern California, Abt Associates, and
Centers for Disease Control and Prevention (CDC)

PLEASE NOTE: It is very important that swab samples are collected as soon as possible. Please collect and return your sample today.

Giving the Swab Sample and Consent Form to Study Staff

STEP 1: Please read and sign the consent form.
STEP 2: Collect your swab sample, following the instructions provided.
STEP 3: Place the sealed zip lock bag into the pre-addressed, postage-paid envelope. Firmly seal the envelope.
STEP 4: Hand the envelope with the collected sample to study staff.
STEP 5: Place the signed consent form in the envelope marked "consent" and hand to study staff.

Mailing/Shipping Instructions for Participants

If you do not want to sign your consent and/or take your swab sample now, please follow the instructions for mailing and shipping the consent and swab sample, below.

[PLEASE READ THIS PAGE FIRST]

Thank you for your participation in the study. In the enclosed kit, you will find instructions and materials for self-collecting a respiratory specimen as well as an **informed consent form**.

Signing and Sending Your Consent Form:

- Only the person who was contacted by telephone and completed the study interview should sign the informed consent form.
- The informed consent form provides details about what will happen in this study. Please read the form carefully and contact Roxana Odouli (Project Manager) at (510) 891-3749 or toll free (888) 381-6818 if you have any questions or concerns about participating in the study.
- Your signatures on the informed consent form are required in order to enroll you in the study. Otherwise the information and nasal swab sample you provided cannot be used. Please sign in ink on the lines where indicated, print your name, and write the date. In order to provide you with your incentive, we will need to receive your signed consent.
- Seal your signed informed consent form in the self-addressed envelope marked "consent form" included with your sample kit. The envelope has pre-paid postage and may be dropped into any U.S. mailbox or left for your mail carrier to pick up.
- You will be given a copy of the consent form for your records.

Swabbing and Sending Your Specimen:

After you have read and signed the informed consent form, please read the **Self-Collection Instructions** and follow them carefully to collect your sample.

- Only the person who was contacted by telephone and completed the study interview should self- collect a respiratory specimen.
- After you have collected a sample, wrap the zip-lock bag in the bubble wrap and place in the box provided.
- Place the box in the UPS laboratory packaging.
- Place the pre-addressed shipping label on the UPS laboratory packaging.
- Please ship the package with your sample on the same day that it was collected. You can either call UPS at 1-800-PICK-UPS (1-800-742-5877) to schedule a pickup, or else you can drop the package off at any UPS drop-off box.

TO VIEW A VIDEO WALK-THROUGH OF THE INSTRUCTIONS, PLEASE SCAN HERE. OR GO TO HTTP://TINYURL.COM/PIPSELFSWABSTAFF

REMINDER: Please do not send the consent form with the swab. The consent form should be mailed separately in the envelope provided.

Please read through all steps before beginning. Call Roxana Odouli (Project Manager) toll free at (888) 381-6818 option 1, if you have any questions about how to perform the nasal swab.

Your Swab Kit Contains:

- These instructions
- Swab
- Transport tube
- Absorbent material (looks like gauze)
- Zip-lock bag
- Pre-addressed, postage-paid envelope
- Bubble wrap
- Sturdy box with lid
- Pre-addressed shipping label and pouch
- UPS laboratory shipping package

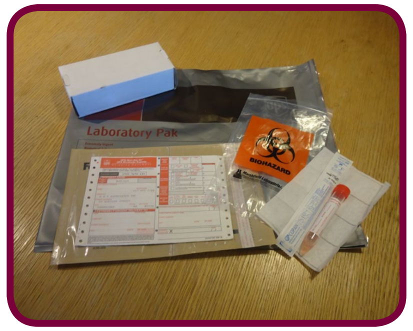

You Will Also Need:

1. A shallow cup or glass

Nasal Swab Self-Collection Instructions

Step by Step Swabbing Instructions:

Step 1

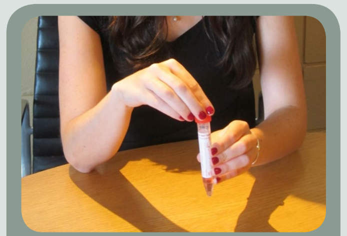

Remove the cap from the transport tube.

Step 2

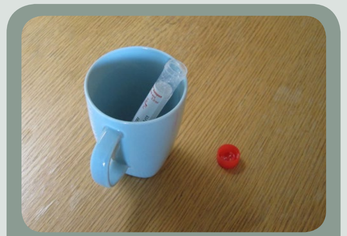

Place the open tube in a shallow cup or glass so that the liquid inside doesn't spill.

Step 3

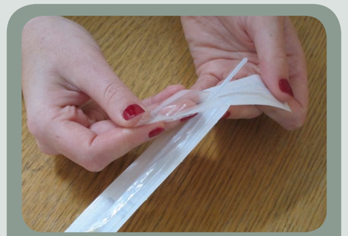

Take the swab out of its package. Be sure not to touch the swab tip. Discard the package.

Step 4

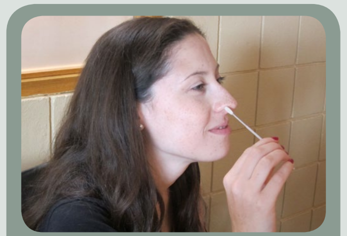

Hold onto the swab's stick about halfway down. Insert the swab into your nostril about half an inch. Rotate the swab inside your nostril, towards the inside of your nose, and circle the swab around your nostril 3 times. While you are circling, keep the swab in contact with your nostril..

Step 5

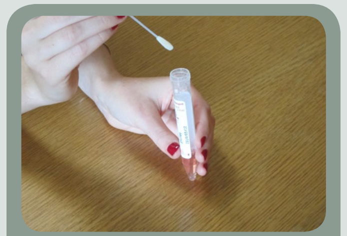

Remove the swab from your nostril. Snap off the end of the swab stick at the break point (look for indentation on the stick). Be sure not to touch the swab tip. Place the swab, swab side down, into the transport tube.

Step 6

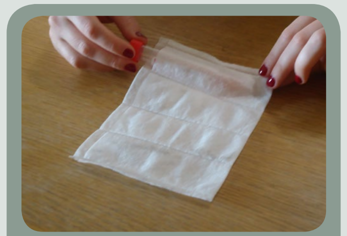

Discard the piece of the stick without the swab end. Twist the cap back on the transport tube, keeping the tube upright. Make sure that the cap is on tight. Slide the tube into the first pocket of the absorbent material.

Step 7

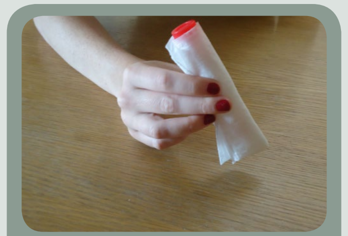

Roll the rest of the material around the tube.

Step 8

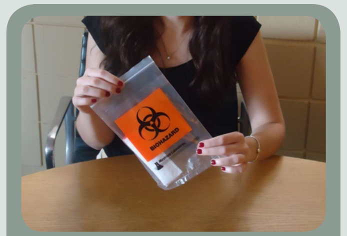

Place the tube, which is now rolled in the absorbent material, into the zip lock bag and seal the bag closed. Wrap the zip-lock bag in the bubble wrap and place in the box provided. Place the box in the UPS laboratory packaging. Place the pre-addressed shipping label on the UPS lab packaging.

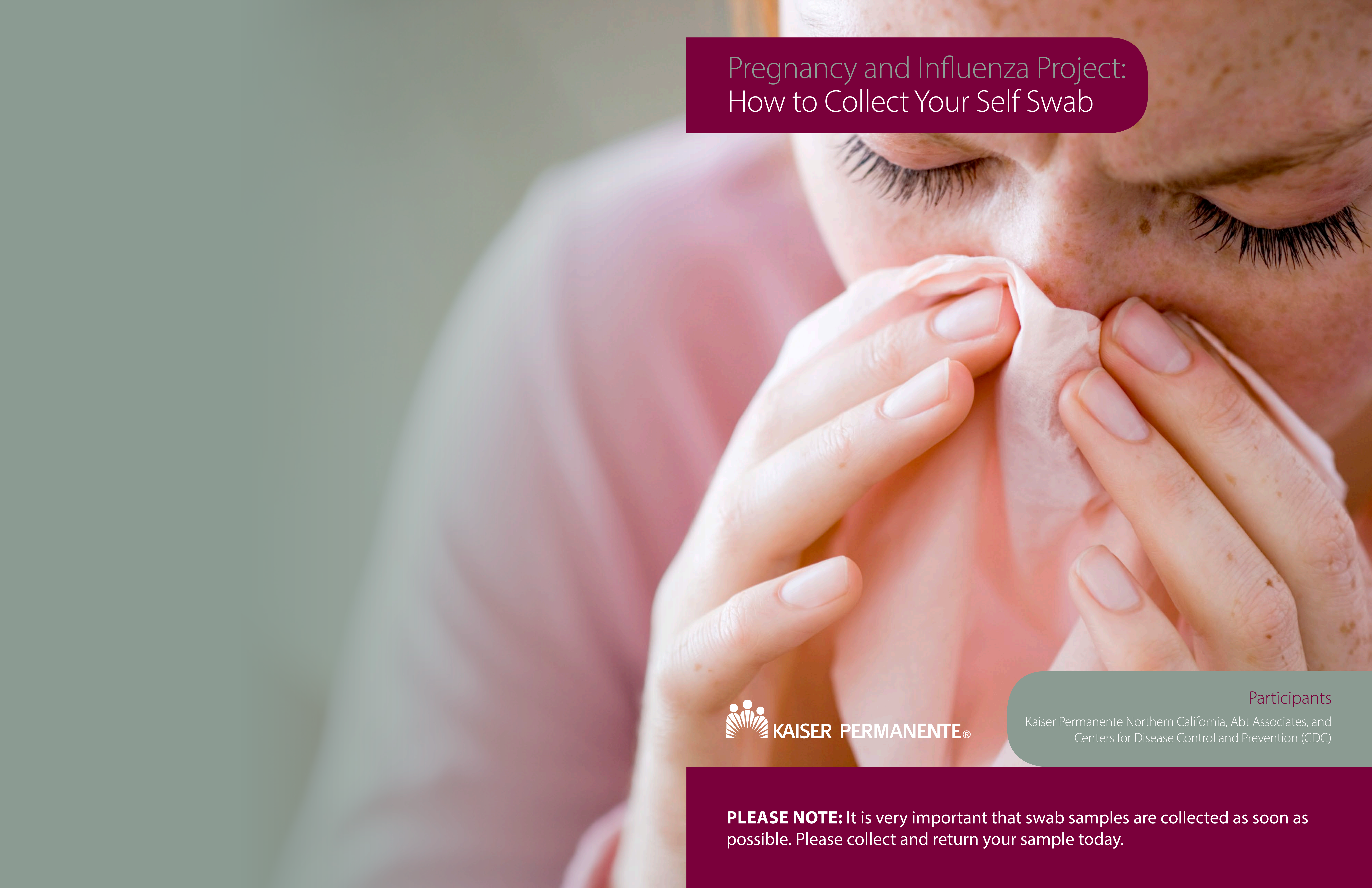

Pregnancy and Influenza Project: How to Collect Your Self Swab

KAISER PERMANENTE®

Participants

Kaiser Permanente Northern California, Abt Associates, and Centers for Disease Control and Prevention (CDC)

PLEASE NOTE: It is very important that swab samples are collected as soon as possible. Please collect and return your sample today.

Mailing/Shipping Instructions for Participants

[PLEASE READ THIS PAGE FIRST]

Thank you for your participation in the study. In the enclosed kit, you will find instructions and materials for self-collecting a respiratory specimen as well as an **informed consent form**.

Signing and Sending Your Consent Form:

- Only the person who was contacted by telephone and completed the study interview should sign the informed consent form.
- The informed consent form provides details about what will happen in this study. Please read the form carefully and contact Roxana Odouli (Project Manager) at (510) 891-3749 or toll free (888) 381-6818 if you have any questions or concerns about participating in the study.
- Your signatures on the informed consent form are required in order to enroll you in the study. Otherwise the information and nasal swab sample you provided cannot be used. Please sign in ink on the lines where indicated, print your name, and write the date. In order to provide you with your incentive, we will need to receive your signed consent.
- Seal your signed informed consent form in the self-addressed envelope marked "consent form" included with your sample kit. The envelope has pre-paid postage and may be dropped into any U.S. mailbox or left for your mail carrier to pick up.
- You will be given a copy of the consent form for your records.

Swabbing and Sending Your Specimen:

After you have read and signed the informed consent form, please read the **Self-Collection Instructions** and follow them carefully to collect your sample.

- Only the person who was contacted by telephone and completed the study interview should self- collect a respiratory specimen.
- After you have collected a sample, wrap the zip-lock bag in the bubble wrap and place in the box provided.
- Place the box in the UPS laboratory packaging.
- Place the pre-addressed shipping label on the UPS laboratory packaging.
- Please ship the package with your sample on the same day that it was collected. You can either call UPS at 1-800-PICK-UPS (1-800-742-5877) to schedule a pickup, or else you can drop the package off at any UPS drop-off box.

TO VIEW A VIDEO WALK-THROUGH OF THE INSTRUCTIONS, PLEASE SCAN HERE OR GO TO HTTP://TINYURL.COM/PIPSELFSWABMAIL

REMINDER: Please do not send the consent form with the swab. The consent form should be mailed separately in the envelope provided.

Please read through all steps before beginning. Call Roxana Odouli (Project Manager) toll free at (888) 381-6818 option 1, if you have any questions about how to perform the nasal swab.

Your Swab Kit Contains:

- These instructions
- Swab
- Transport tube
- Absorbent material (looks like gauze)
- Zip-lock bag
- Pre-addressed, postage-paid envelope
- Bubble wrap
- Sturdy box with lid
- Pre-addressed shipping label and pouch
- UPS laboratory shipping package

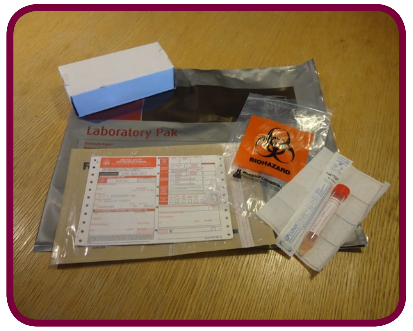

You Will Also Need:

1. A shallow cup or glass

Nasal Swab Self-Collection Instructions

Step by Step Swabbing Instructions:

Step 1

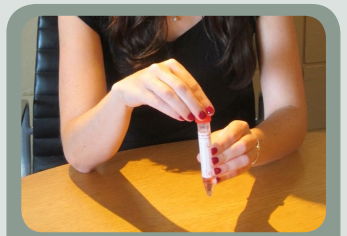

Remove the cap from the transport tube.

Step 2

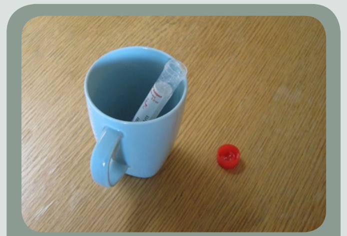

Place the open tube in a shallow cup or glass so that the liquid inside doesn't spill.

Step 3

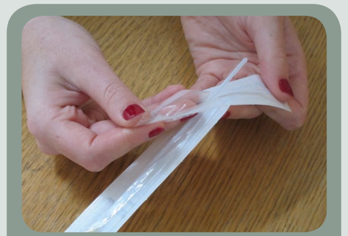

Take the swab out of its package. Be sure not to touch the swab tip. Discard the package.

Step 4

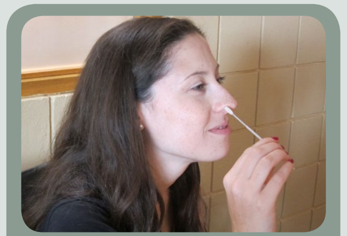

Hold onto the swab's stick about halfway down. Insert the swab into your nostril about half an inch. Rotate the swab inside your nostril, towards the inside of your nose, and circle the swab around your nostril 3 times. While you are circling, keep the swab in contact with your nostril..

Step 5

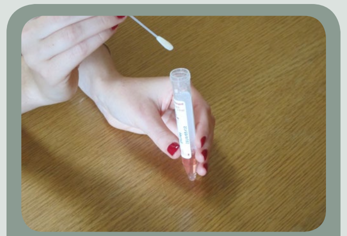

Remove the swab from your nostril. Snap off the end of the swab stick at the break point (look for indentation on the stick). Be sure not to touch the swab tip. Place the swab, swab side down, into the transport tube.

Step 6

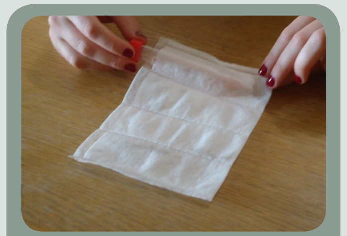

Discard the piece of the stick without the swab end. Twist the cap back on the transport tube, keeping the tube upright. Make sure that the cap is on tight. Slide the tube into the first pocket of the absorbent material.

Step 7

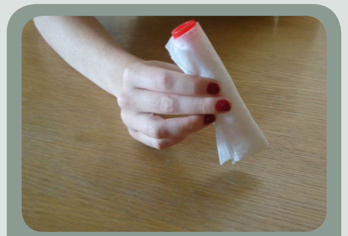

Roll the rest of the material around the tube.

Step 8

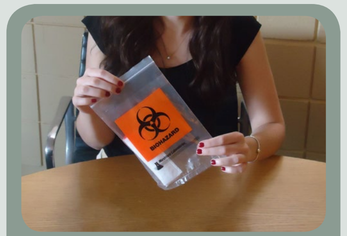

Place the tube, which is now rolled in the absorbent material, into the zip lock bag and seal the bag closed. Wrap the zip-lock bag in the bubble wrap and place in the box provided. Place the box in the UPS laboratory packaging. Place the pre-addressed shipping label on the UPS lab packaging.
